# DDX3 depletion represses translation of mRNAs with complex 5′ UTRs

**DOI:** 10.1093/nar/gkab287

**Published:** 2021-04-27

**Authors:** Lorenzo Calviello, Srivats Venkataramanan, Karol J Rogowski, Emanuel Wyler, Kevin Wilkins, Malvika Tejura, Bao Thai, Jacek Krol, Witold Filipowicz, Markus Landthaler, Stephen N Floor

**Affiliations:** Department of Cell and Tissue Biology, University of California, San Francisco, San Francisco, CA 94143, USA; Department of Cell and Tissue Biology, University of California, San Francisco, San Francisco, CA 94143, USA; Berlin Institute for Medical Systems Biology, Max-Delbrück-Center for Molecular Medicine in the Helmholtz Association, 13125 Berlin, Germany; Berlin Institute for Medical Systems Biology, Max-Delbrück-Center for Molecular Medicine in the Helmholtz Association, 13125 Berlin, Germany; Department of Cell and Tissue Biology, University of California, San Francisco, San Francisco, CA 94143, USA; Department of Cell and Tissue Biology, University of California, San Francisco, San Francisco, CA 94143, USA; Department of Cell and Tissue Biology, University of California, San Francisco, San Francisco, CA 94143, USA; Institute of Molecular and Clinical Ophthalmology Basel, Basel, Switzerland; Friedrich Miescher Institute for Biomedical Research, Basel, Switzerland; Berlin Institute for Medical Systems Biology, Max-Delbrück-Center for Molecular Medicine in the Helmholtz Association, 13125 Berlin, Germany; IRI Life Sciences, Institut für Biologie, Humboldt Universität zu Berlin, Philippstraße 13, 10115 Berlin, Germany; Department of Cell and Tissue Biology, University of California, San Francisco, San Francisco, CA 94143, USA; Helen Diller Family Comprehensive Cancer Center, University of California, San Francisco, San Francisco, CA 94143, USA

## Abstract

DDX3 is an RNA chaperone of the DEAD-box family that regulates translation. Ded1, the yeast ortholog of DDX3, is a global regulator of translation, whereas DDX3 is thought to preferentially affect a subset of mRNAs. However, the set of mRNAs that are regulated by DDX3 are unknown, along with the relationship between DDX3 binding and activity. Here, we use ribosome profiling, RNA-seq, and PAR-CLIP to define the set of mRNAs that are regulated by DDX3 in human cells. We find that while DDX3 binds highly expressed mRNAs, depletion of DDX3 particularly affects the translation of a small subset of the transcriptome. We further find that DDX3 binds a site on helix 16 of the human ribosomal rRNA, placing it immediately adjacent to the mRNA entry channel. Translation changes caused by depleting DDX3 levels or expressing an inactive point mutation are different, consistent with different association of these genetic variant types with disease. Taken together, this work defines the subset of the transcriptome that is responsive to DDX3 inhibition, with relevance for basic biology and disease states where DDX3 is altered.

## INTRODUCTION

Translation initiation is affected by mRNA regulatory elements. The DEAD-box RNA chaperone DDX3 and its yeast ortholog Ded1 facilitate translation initiation on mRNAs with structured 5′ untranslated regions (UTRs) (1–4), a function that is essential in all eukaryotes (5). Dysfunction in DDX3 is linked to numerous diseases, including medulloblastoma (3,6–12), many other cancer types (5) and *de novo* developmental delay (13–16). Previous work studied how translation is altered by DDX3 variants found in medulloblastoma (3,12), which are exclusively missense variants that preferentially target conserved residues. In contrast, hematological cancers like natural killer/T-cell lymphoma (17,18) and others (19,20) also have frequent variants in *DDX3X*, but they are mostly truncating or frameshift variants resulting in decreased expression. Changes in gene expression occurring as a result of decreased DDX3 levels remain incompletely understood.

Inactivation of Ded1 in yeast leads to polysome collapse and global downregulation of translation (21,22). More recent work showed that Ded1 is required for translation of most transcripts in yeast using genome-wide approaches (1,23). In contrast, DDX3 depletion seems to only affect translation of a subset of expressed transcripts (2,4,24–26). Despite the importance of DDX3 to normal function and its alteration in diverse disease states, the set of genes that depend on DDX3 for translation is not clearly defined. Moreover, it has been challenging to relate DDX3 binding to functional effects on bound mRNAs, and it was unclear if DDX3 functions outside of translation initiation given that binding was detected in coding sequences and 3′ UTRs (3,12).

Here, we depleted DDX3 protein levels and measured alterations to translation and RNA abundance using ribosome profiling and RNA-seq. We also characterized DDX3 binding by PAR-CLIP, using the presence of T>C mutations as a diagnostic hallmark of protein–RNA interactions. We observed robust interactions between DDX3 and transcript 5′ UTRs, as well as a specific and conserved site on the 18S ribosomal rRNA. We found that transcripts with structured 5′ UTRs are preferentially affected by DDX3. We used *in vitro* and cellular reporter systems to conclude that decreases in ribosome occupancy upon DDX3 depletion are driven by 5′ UTRs. Taken together, our results support a model for DDX3 function where interactions with the small ribosomal subunit facilitate translation on messages with structured 5′ UTRs, which, when inactivated, pathologically deregulates protein synthesis.

## MATERIALS AND METHODS

### NGS data pre-processing

Ribo-seq fastq files were stripped of the adapter sequences using cutadapt. UMI sequences were removed and reads were collapsed to fasta format. Reads were first aligned against rRNA (accession number U13369.1), and to a collection of snoRNAs, tRNAs and miRNA (retrieved using the UCSC table browser) using bowtie2 (27) in the ‘local’ alignment mode.

Remaining reads were mapped to the hg38 version of the genome (without scaffolds) using STAR 2.6.0a (28) supplied with the GENCODE 32 .gtf file. A maximum of three mismatches and mapping to a maximum of 50 positions was allowed. *De-novo* splice junction discovery was disabled for all datasets. Only the best alignment per each read was retained. Read counts for all libraries are in Supplementary Table S3.

### PAR-CLIP peak calling

Peak calling for PAR-CLIP reads was performed with PARalyzer v1.5 (29) in the ‘EXTEND_BY_READ’ mode using the following parameters:

BANDWIDTH = 3
CONVERSION = T>C
MINIMUM_READ_COUNT_PER_GROUP = 5
MINIMUM_READ_COUNT_PER_CLUSTER = 5
MINIMUM_READ_COUNT_FOR_KDE = 5
MINIMUM_CLUSTER_SIZE = 8
MINIMUM_CONVERSION_LOCATIONS_FOR_CLUSTER = 1
MINIMUM_CONVERSION_COUNT_FOR_CLUSTER = 3
MINIMUM_READ_COUNT_FOR_CLUSTER_INCLUSION = 5
MINIMUM_READ_LENGTH = 18
MAXIMUM_NUMBER_OF_NON_CONVERSION_MISMATCHES = 0
Peaks with >10 reads were retained for subsequent analysis.

Coverage-normalized T>C conversions on rRNA for positions with 2000 reads or more (Supplementary Figure S4A) were mapped onto the 18S rRNA sequence from PDB entry 6FEC and visualized using UCSF Chimera (30).

### Differential expression analysis

Count matrices for Ribo-seq and RNA-seq were built using reads mapping uniquely to CDS regions of protein-coding genes, using the Bioconductor packages *GenomicFeatures*, *GenomicFiles* and *GenomicAlignments* (31). Genomic and transcript regions where extracted using *Ribo-seQC* (32). Only reads mapping for more than 25nt were used.

Differential analysis was using *DESeq2* (33). Concordant changes were defined using an FDR cutoff of 0.01 for RNA-seq and Ribo-seq individually and ensuring the same directionality in the estimated fold changes.

Changes in translation efficiency were calculated using *DESeq2* by using assay type (RNA-seq or Ribo-seq) as an additional covariate. Translationally regulated genes were defined using an FDR cutoff of 0.05 from a likelihood ratio test, using a reduced model without the assay type covariate, e.g. assuming no difference between RNA-seq and Ribo-seq counts (34).

For both RNA-seq and Ribo-seq, only genes with BaseMean >8 or more than the bottom 10% of the library were used. GO enrichment analysis was performed with the *topGO* package (version 2.38.1; available from BioConductor), using the Fisher test with default parameters.

The Random Forest regression was run using the *randomForest* package (version 4.6-14; available from CRAN) with default parameters. Lasso regression was performed on scaled variables using the *glmnet* package (35). The following features for each gene were used:

- TPM values using RNA-seq (in log scale);
- Baseline TE levels, defined as ratio of Ribo to RNA reads;
- Baseline RNA mature levels, defined as length-normalized ratio of RNA-seq reads in introns versus exons;
- GC content, length (in log scale) and ribosome density in: 5′ UTRs, a window of 25nt around start and stop codons, CDS regions, non-coding internal exons, introns, and 3′ UTRs;
- Additional sequence features, including density of motif scores (calculated using the *Biostrings* package) for the following motifs, partially taken from (36):

- TOP: a binary variable indicating if gene belongs to core TOP mRNA, as defined in (37)
- PRTE(pyrimidine-rich translational element): ‘[CU][CU][CU][CU][U][CU][CU][CU]’
- TISU(Translator Initiator of Short 5′-UTR): ‘[CG][A][A][CG][A][U][G][G][C][G][G][C]’
- CERT (cytosine-enriched regulator of translation): ‘[CG][CGU][CGU][CG][CGU][C][CGU][C][CA][GU][C][CGU][CGUA][CG][C]’
- PQS: propensity to create G quadruplexes, calculated using the *pqsfinder* R package (38)

Feature importance (measured by mean decrease in accuracy for the random forest model) and correlation between predicted and measured test data were calculated on a 10-fold cross-validation scheme.

### Meta-transcript profiles

PARalyzer peaks (and peaks from the POSTAR2 repository (39)) were mapped on transcript coordinates using one coding transcript per gene: such transcript was chosen to have the longest 5′ UTR and the most common annotated start codon for that gene. Transcript positions were converted into bins using 15 bins for each 5′ UTR, 30 bins for each CDS and 20 bins for each 3′ UTR. Peak scores were normalized for each transcript (to sum up to 1), and values were summed for each bin to build aggregate profiles, as in Figure 4B. When plotting profiles for different RBPs. the aggregate profiles were further normalized to a sum of 1. To build the average meta-transcript profile in Figure 4C, conversion specificity values were averaged per transcript bin. To create shuffled profiles in Figure 4F and Supplementary Figure S4, 5 random positions for each peak were taken from the same bound UTR.

### De novo motif finding

The STREME algorithm (40) was used to perform *de novo* motif finding, using PAR-CLIP peaks from the POSTAR2 repository, selecting peaks from HEK 293 cells called with PARalyzer as above, and selecting for peaks in UTRs and intronic regions in protein-coding genes. The following parameters were used:

–rna –minw 5 –maxw 15 –pvt 0.05 –totallength 1e7 –time 18000 –patience 6

### Additional transcript features analysis

To compare read mapping locations within transcripts, a window of 25nt around the start codon was subtracted from annotated 5′ UTRs and CDS. 5′ UTRs and CDS regions in genomic and transcriptomic space were retrieved using *Ribo-seQC*. Counts on 5′ UTR and CDS were first averaged between replicates. The ratio 5′ UTR to CDS of these counts were calculated for each gene, in the siRNA and controls condition. The log_2_ of the ratio siDDX3/control for those values represents the skew of counts towards 5′ UTR in the siDDX3 condition:

$$\begin{eqnarray*}&&5^{\prime}UTR\;ribosome\;skew\;\\ &&\quad= lo{g_2}\;\left[ {{{\left( {\frac{{R{P^{5^{\prime}UTR}}}}{{R{P^{CDS}}}}} \right)}_{siDDX3}}/{{\left( {\frac{{R{P^{5^{\prime}UTR}}}}{{R{P^{CDS}}}}} \right)}_{control}}} \right] \end{eqnarray*}$$

RNA *in silico* folding was performed on 5′ UTRs sequences using RNAlfold (41) with default parameters. Average ΔG values per nucleotide were calculated averaging the ΔG values of each structure overlapping that nucleotide. %GC content and T>C transition specificity (defined as *ConversionEventCount* / (*ConversionEventCount* + *NonConversionEventCount*)) for each PAR-CLIP peak were derived using the clusters.csv output file from PARalyzer. *Gviz* was used to plot tracks for RNA-seq, Ribo-seq and PAR-CLIP over different transcripts. The Wilcoxon rank sum was used for statistical testing and Cliff's delta was used to calculate effect sizes as described (42).

Source code to reproduce figures can be found at:

https://github.com/lcalviell/DDX3X_RPCLIP

### Ribosome profiling

Knockdown RP: Flp-In T-REx HEK 293 cells transfected with control siPool and with *DDX3X*-targeting siPools (two replicates each, siTOOLs Biotech; sequences in Supplementary Table S5) were washed with PBS containing 100 μg/ml cycloheximide to trap ribosomes, flash frozen on liquid nitrogen, lysed in lysis buffer (20 mM Tris–HCl pH 7.4, 150 mM NaCl, 5 mM MgCl_2_ 1% (v/v) Triton X-100, 25 U/ml TurboDNase (Ambion), harvested, centrifuged at 20 000 g for 4 min at 4°C and supernatants were stored at –80°C. Thawed lysates were treated with RNase I (Ambion) at 2.5 U/μl for 45 min at room temperature with slow agitation. Further RNase activity was stopped by addition of SUPERase:In (Ambion). Next Illustra MicroSpin Columns S-400 HR (GE Healthcare) were used to enrich for ribosome complexes. RNA was extracted from column flow throughs with TRIzol (Ambion) reagent. Precipitated nucleic acids were further purified and concentrated with Zymo-Spin IIC column (Zymo Research). Obtained RNA was depleted of rRNAs with Ribo-Zero Gold Kit (Human/Mouse/Rat) kit (Illumina), separated in 17% urea gel and stained with SYBR Gold (Invitrogen). Gel slices containing nucleic acids 27–30 nucleotides long were excised and incubated in a thermomixer with 0.3 M NaCl at 4°C overnight with constant agitation to elute RNA. After precipitation nucleic acids were treated with T4 polynucleotide kinase (Thermo Scientific). Purified RNA was ligated to 3′ and 5′ adapters, reverse transcribed and PCR amplified. The amplified cDNA was sequenced on a HiSeq2000 (Illumina).

Degron RP: *DDX3X*-mAID tagged HCT 116 (one 15 cm dish at 80–90% confluency per replicate, two replicates) cells expressing OsTIR1 were transfected with either wild-type *DDX3X* or *DDX3X* R326H. 24 hours post-transfection, media was changed and fresh media with 500 μM indole 3-acetic acid (IAA) was added to cells. Un-transfected cells were treated with either DMSO or IAA. Forty-eight hours after auxin addition, cells were treated with 100 μg/ml cycloheximide (CHX) for two minutes to trap ribosomes and harvested and lysed as described in (43). Briefly, cells were washed with PBS containing 100 μg/ml CHX and lysed in ice-cold lysis buffer (20 mM Tris–HCl pH 7.4, 150 mM NaCl, 5 mM MgCl_2_, 1 mM DTT, 100 μg/ml CHX, 1% (v/v) Triton X-100, 25 U/ml TurboDNase (Ambion). 240 μl lysate was treated with 6 μl RNase I (Ambion, 100 U/μl) for 45 min at RT with gentle agitation and further digestion halted by addition of SUPERase:In (Ambion). Illustra Microspin Columns S-400 HR (GE healthcare) were used to enrich for monosomes, and RNA was extracted from the flow-through using Direct-zol kit (Zymo Research). Gel slices of nucleic acids between 24 and 32 nts long were excised from a 15% urea–PAGE gel. Eluted RNA was treated with T4 PNK and preadenylated linker was ligated to the 3′ end using T4 RNA Ligase 2 truncated KQ (NEB, M0373L). Linker-ligated footprints were reverse transcribed using Superscript III (Invitrogen) and gel-purified RT products circularized using CircLigase II (Lucigen, CL4115K). rRNA depletion was performed using biotinylated oligos as described in (44) and libraries constructed using a different reverse indexing primer for each sample.

### PAR-CLIP experiments

Flp-In T-REx HEK 293 cells expressing FLAG/HA-tagged *DDX3X* (45) were labeled with 100 μM 4-thiouridine for 16h. PAR-CLIP was performed generally as described (46,47). Briefly, cells were UV-crosslinked with 0.15 J/cm^2^ at 365 nm, and stored at –80°C. Obtained cell pellets were lysed in three times the cell pellet volume of NP-40 lysis buffer (50 mM HEPES-KOH at pH 7.4, 150 mM KCl, 2 mM EDTA, 1 mM NaF, 0.5% (v/v) NP-40, 0.5 mM DTT, complete EDTA-free protease inhibitor cocktail (Roche)), incubated 10 min on ice and centrifuged at 13000 rpm for 15 min at 4°C. Supernatants were filtered through 5 μm syringe filter. Next, lysates were treated with RNase I (Thermo-Fisher Scientific) at final concentration of 0.25 U/μl for 10 min at room temperature. Immunoprecipitation of the DDX3/RNA complexes was performed with FLAG magnetic beads (Sigma). After IP and washing, the protein-bound RNAs were 3′ de-phosphorylated and 5′-end phosphorylated using T4 PNK with 0.01% Triton X-100, and the NIR fluorescent adaptor (5′-OH-AGATCGGAAGAGCGGTTCAGAAAAAAAAAAAA/iAzideN/AAAAAAAAAAAA/3Bio/-3′) was ligated to the RNA using truncated RNA ligase 2 K227Q (NEB) overnight at 16°C, shaking at 1600 rpm. Crosslinked protein–RNA complexes were resolved on a 4–12% NuPAGE gel (Thermo-Fisher Scientific) and transferred to a nitrocellulose membrane. Protein–RNA complex migrating at an expected molecular weight were excised, and RNA by proteinase K (Roche) treatment and phenol–chloroform extraction. Purified RNA was further ligated to 5′ adapters, reverse transcribed and PCR amplified. The amplified cDNA was sequenced on a NextSeq 500 device (Illumina).

### *In vitro* transcription, capping, and 2′-O methylation of reporter RNAs

Annotated 5′ UTRs for selected transcripts were cloned upstream of Renilla Luciferase (RLuc) under the control of a T7 promoter, with 60 adenosine nucleotides downstream of the stop codon to mimic polyadenylation. 5′ UTR sequences are in Supplementary Table S4. Untranslated regions were cloned using synthetic DNA (Integrated DNA Technologies) or by isolation using 5′ RACE (RLM-RACE kit, Invitrogen). Template was PCR amplified using Phusion polymerase from the plasmids using the following primers, and gel purified, as described (42).

pA60 txn rev: TTT TTT TTT TTT TTT TTT TTT TTT TTT TTT TTT TTT TTT TTT TTT TTT TTT TTT TTT TTT CTG CAG

pA60 txn fwd: CGG CCA GTG AAT TCG AGC TCT AAT ACG ACT CAC TAT AGG

100 μl *in vitro* transcription reactions were set up at room temperature with 1–5 μg of purified template, 7.5 mM ACGU ribonucleotides, 30 mM Tris–Cl pH 8.1, 125 mM MgCl_2_, 0.01% Triton X-100, 2 mM spermidine, 110 mM DTT, T7 polymerase and 0.2 U/μl units of Superase-In RNase inhibitor (Thermo-Fisher Scientific). Transcription reactions were incubated in a PCR block at 37°C for 1 h. 1 μl of 1 mg/ml pyrophosphatase (Roche) was added to each reaction, and the reactions were subsequently incubated in a PCR block at 37°C for 3 h. 1 unit of RQ1 RNase-free DNase (Promega) was added to each reaction followed by further incubation for 30 minutes. RNA was precipitated by the addition of 200 μl 0.3 M NaOAc pH 5.3, 15 μg GlycoBlue co-precipitant (Thermo-Fisher Scientific) and 750 μl 100% EtOH. Precipitated RNA was further purified over the RNA Clean & Concentrator-25 columns (Zymo Research). Glyoxal gel was run to assess the integrity of the RNA before subsequent capping and 2′ *O*-methylation.

20 μg of total RNA was used in a 40 μl capping reaction with 0.5mM GTP, 0.2 mM *S*-adenosylmethionine (SAM), 20 units of Vaccinia capping enzyme (New England Biolabs), 100 units of 2′-*O*-Me-transferase (New England Biolabs) and 25 units RNasin Plus RNase inhibitor (Promega). The reactions were incubated at 37°C for 1 h, followed by purification over the RNA Clean & Concentrator-25 columns (Zymo Research) and elution in DEPC H_2_O. Glyoxal gel was run to assess the integrity of the RNA before proceeding to *in vitro* translation reactions.

### Transfection of siRNA for *in vitro* translation

HEK 293T cells in 150mM plates were transfected with 20 μl of siRNA (against DDX3 or a non-targeting control; sequences in Supplementary Table S5) using Lipofectamine 2000 (Thermo Fisher Scientific), following manufacturer's instructions. Cells were harvested for preparation of cellular extracts after 48 h.

### Generation of DDX3 mutant translation extracts

DDX3 WT and R326H mutant constructs were synthesized and cloned downstream of a CMV promoter (Twist Biosciences). 40 μg of plasmids were transfected into HCT 116 cells using Lipofectamine 2000 (Thermo Fisher Scientific), following manufacturer's instructions. Cells were treated with 500 μM indole-3-acetic acid (IAA) 24 h post-transfection and harvested for preparation of cellular extracts after a further 48 h.

### Preparation of cellular extracts for *in vitro* translation

Three to five 150 mm plates of HEK 293T or HCT 116 cells were trypsinized and pelleted at 1000g, 4°C. One cell-pellet volume of lysis buffer (10 mM HEPES, pH 7.5, 10 mM KOAc, 0.5 mM MgOAc_2_, 5 mM DTT, and 1 tablet Complete mini EDTA free protease inhibitor (Sigma) per 10 ml) was added to the cell pellet and was incubated on ice for 45 min. The pellet was homogenized by trituration through a 26G needle attached to a 1 ml syringe 13–15 times. Efficiency of disruption was checked by trypan blue staining (>95% disruption target). The lysate was cleared by centrifugation at 14000g for 1 min at 4°C, 2–5 μl was reserved for western blot analysis, and the remainder was aliquoted and flash frozen in liquid nitrogen.

### Antibodies

**Figure 1.**
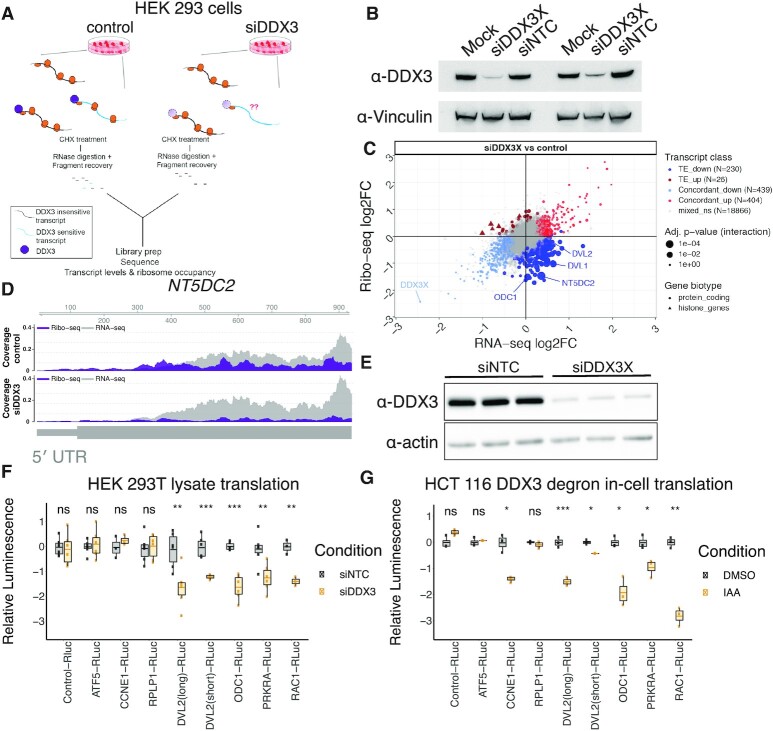
Translational changes upon DDX3 depletion. (**A**) A workflow of the ribosome profiling experiments. (**B**) siRNA knockdown efficiency of DDX3 analyzed by western blot. Mock: untreated. NTC: nontargeting control siRNA. (**C**) The log_2_ fold change (log_2_FC) in ribosome profiling or RNA levels are plotted for all genes. Number genes in each category are indicated with selected genes labeled. Point size indicates the *P*-value of a significant change in translational efficiency. (**D**) Tracks showing RNA-seq (RNA) or ribosome profiling (RP) reads that map to the *NT5DC2* mRNA. *NT5DC2* is an mRNA with differential ribosome occupancy in (C). (**E**) Western blot analysis of nontargeting (siNTC) or siDDX3 translation lysate samples with antibodies indicated. (**F**) Renilla luciferase luminescence from *in vitro* transcribed reporter RNAs translated *in vitro* in siNTC or siDDX3 HEK 293T lysates. (**G**) Reporter RNAs as in (F) transfected into HCT 116 cells with or without IAA treatment to induce the DDX3 degron.

Primary antibodies used in this study include anti-DDX3 (Bethyl A300-474A; Figure 1), rabbit polyclonal anti-DDX3 (custom made by Genemed Synthesis using peptide ENALGLDQQFAGLDLNSSDNQS; Figure 5; (26)), anti-actin HRP (Santa Cruz Biotechnology, sc-47778), anti-FLAG HRP (Sigma, A8592).

### *In vitro* translation

5 μl *in vitro* translation reactions were set up with 2.5 μl of lysate and 20 ng total RNA (0.84 mM ATP, 0.21 mM GTP, 21 mM creatine phosphate, 0.009 units/ml creatine phosphokinase, 10 mM HEPES pH 7.5, 2 mM DTT, 2 mM MgOAc, 100 mM KOAc, 0.008 mM amino acids, 0.25 mM spermidine, 5 units RNasin Plus RNase inhibitor (Promega) as described (48). Reaction tubes were incubated at 30°C for 45 min, and expression of the reporter was measured using the Renilla Luciferase Assay System (Promega) on a GloMax Explorer plate reader (Promega).

### In cell translation

*DDX3X*-mAID tagged HCT-116 cells expressing OsTIR1 were plated into 96-well plates at ∼80% confluency. Cells were pre-treated with DMSO or 500 μM indole 3-acetic acid (IAA) for 48 h. Cells were transfected with a mixture of 10 ng *in vitro* transcribed firefly luciferase RNA with a minimal 5′ UTR (FLuc) and between 50–200 ng renilla luciferase RNA (RLuc) downstream of selected 5′ UTRs using the TransIT mRNA Transfection Kit (Mirus Bio) per manufacturer's instructions. Twenty-four hours post transfection, cells were lysed and expression of the reporters were measured using the Dual-Luciferase Reporter Assay System (Promega) on a GloMax Explorer plate reader (Promega). The Renilla Luciferase signal in each well was normalized to the Firefly Luciferase signal to control for cell number, transfection efficiency, and basal translation levels.

## RESULTS

### Identifying mRNAs that depend on DDX3 for efficient translation

We performed ribosome profiling and RNA-seq to determine the set of transcripts that are affected by depletion of DDX3. *DDX3X* is an essential gene (26,49), so we transiently knocked down its expression using siRNA and collected ribosome protected footprints in duplicate experiments (Figure 1A, B, Supplementary Figure S1A). Knockdown efficiencies were ∼90% and ∼70% in replicates (Figure 1B). Measuring changes in both RNA abundance and ribosome occupancy enabled us to distinguish between different modes of DDX3-mediated regulation. We found that depletion of DDX3 affects ribosome occupancy of a minority of messages (Figure 1C). Most changes in ribosome occupancy upon DDX3 depletion were decreases, broadly suggesting that the function of DDX3 is to increase ribosome occupancy. Genes such as *DVL2, NT5DC2* and *ODC1*, which is described to be translationally-controlled (50), decreased in ribosome occupancy upon DDX3 depletion (Figure 1C, D, Supplementary Table S1). Diverse biological pathways were affected by DDX3 depletion, revealing the enrichment of histone mRNAs among the few examples of translationally upregulated transcripts, which might reflect a resistance to a widespread translation suppression rather than an increase in protein synthesis. Genes related to neuronal branching belonged to the translationally downregulated set (Supplementary Figure S1B). To confirm the effects of DDX3 depletion on the translated transcriptome, we also established a cell line to rapidly and efficiently degrade endogenous DDX3 in human male-derived colorectal cancer HCT 116 cells, a near-diploid cell line amenable to genome engineering, upon addition of auxin (Supplementary Figure S2A; (51). As with the siRNA knockdown, induced degradation of DDX3 predominantly resulted in a marked decrease in the translation of a subset of cellular messages (Supplementary Figure S2B). Translation efficiency (TE) changes were more similar than steady state RNA levels upon siRNA knockdown or chemical degradation (Supplementary Figure S2C, Supplementary Table S1), even though these experiments were performed in different cell lines (HEK 293T versus HCT 116) and with different depletion approaches, affirming DDX3 function in translation regulation.

Our data suggest that DDX3 directly affects translation of a subset of mRNAs. However, ribosome profiling measures ribosome density, which can be affected by changes to translation initiation, translation elongation, or ribosome stalling. DDX3 is thought to regulate translation through transcript 5′ UTRs, and we found genes that are regulated by their 5′ UTRs such as *ODC1* in the translationally downregulated set (Figure 1C; (50,52). To test whether altered translation initiation contributes to the impact of DDX3 knockdown on ribosome density, we cloned DDX3-sensitive 5′ UTRs from this and previous work (3,25,53) upstream of a *Renilla* luciferase reporter and compared them to a control reporter that is not sensitive to DDX3 depletion. Since *DVL2* has many annotated 5′ UTRs that overlap, we cloned the prevalent isoforms in HEK 293T cells using 5′ RACE, which yielded a short and long isoform (Materials and Methods). We then made translation extracts from HEK 293T cells transfected with a nontargeting control siRNA or a DDX3 siRNA (Figure 1E). Next, reporter RNAs were *in vitro* transcribed, capped, and 2′-O methylated and used for *in vitro* translation in wild-type or DDX3-depleted lysate. We found that the 5′ UTRs from the DDX3-sensitive mRNAs *ODC1*, *PRKRA*, *RAC1* and *DVL2* also conferred DDX3 dependence to the luciferase reporter (Figure 1F). However, other reporter RNAs, such as *ATF5* or and *RPLP1*, did not change in the *in vitro* translation upon DDX3 knockdown. *RPLP1* was identified as an mRNA with uORF occupancy changes upon mutant DDX3 expression in prior work (3), while *ATF5* was previously implicated in DDX3-dependent translation (25,53). To compare *in vitro* translation with translation in cellular contexts, we transfected the same reporter mRNAs into HCT 116 cells with DDX3 depleted using our degron system (Figure 1G, Supplementary Figure S2D). The results of the *in vitro* and cellular translation assays show a high degree of concordance, with the exception of the *CCNE1* 5′UTR, which was previously shown to require DDX3 for efficient translation (25). Therefore, based on these reporter experiments, we interpret ribosome occupancy changes upon DDX3 depletion as a result of mis-regulated translation initiation dynamics and refer to them as translation efficiency (TE).

### Defining the features that mediate DDX3-dependent translation

**Figure 2.**
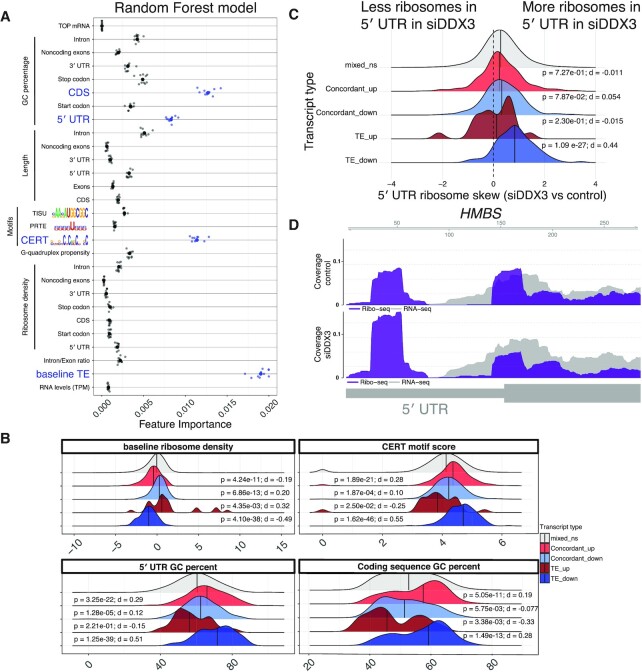
DDX3-sensitive transcripts have complex 5′ leaders. (**A**) Strength of individual random forest model features that predict TE with *R* = 0.54 (Supplementary Figure S2A, Materials and Methods). Features in blue are plotted in (B). (**B**) The translation efficiency (TE) in wild-type cells, CERT motif score, GC-content of 5′ UTRs, and GC content of coding sequences in the indicated gene sets from Figure 1C were computed and are plotted as a density plot based on their importance in the random forest model. Vertical lines are medians. *P*-values for Wilcoxon rank sum and effect sizes from Cliff's delta (*d*) versus mixed_ns indicated. (**C**) The fold-change of the ratio in ribosome occupancy in the 5′ UTR versus the coding sequence upon DDX3 depletion as a density plot for transcripts in the indicated sets. A larger 5′ UTR skew value means that there are more ribosomes in the 5′ UTR compared to the coding sequence. P-values from Wilcoxon rank sum and effect sizes from Cliff's delta (d) versus mixed_ns indicated. (**D**) An example gene (HMBS) with increases in 5′ UTR ribosomes versus coding sequence ribosomes with tracks as in Figure 1D.

The *in vitro* and cellular translation experiments implicated translation initiation and transcript 5′ UTRs in mRNAs that are sensitive to DDX3 depletion. To determine which features contribute to quantitative changes in translation upon knockdown of DDX3, we used known translational-control elements to generate a random forest regression model. A model with 28 features (Materials and Methods) was able to moderately predict the translation changes upon DDX3 knockdown (correlation between predicted and observed changes = 0.54, Supplementary Figure S3A), with few features driving the model performance (Figure 2A, Materials and Methods), such as baseline translation levels, GC content in coding sequences and 5′ UTRs, and density of the CERT motif. The CERT motif is a cytosine-rich element that has been implicated in eIF4E- and eIF4A-dependent translation through incompletely understood mechanisms (54,55). Interestingly, mRNAs that are sensitive to DDX3 depletion appear to be poorly translated in HEK 293T cells (Figure 2B). A reduced random forest regression model only using the four most relevant features performed similarly; conversely, a model built without using sequence predictors or baseline translation levels could not recapitulate translation downregulation effects (Supplementary Figure S3B, S3C). 5′ UTR and coding sequence GC content may be indications of increased RNA structure in these regions, possibly relevant for other aspects of cytoplasmic RNA processing (Discussion).

To test the ability of the random forest to select relevant features predictive of DDX3-mediated translation changes, we compared it to lasso regression, another method used to perform feature selection among a set of correlated predictors (Materials and Methods). The two methods largely agreed in pinpointing relevant features, with the random forest slightly outperforming the lasso (Supplementary Figure S3D, E). The features predicted to drive DDX3 sensitivity also showed different distributions among the regulated mRNAs, especially in the translationally downregulated set, indicating that the features identified by the random forest are indeed different between sets of transcripts (Figure 2B).

We measured the enrichment of ribosomes in transcript 5′ UTRs, under the hypothesis that depletion of DDX3 might lead to defective scanning and ribosome accumulation (1), or selective ribosome depletion on coding sequences. Indeed, we found more ribosomes in transcript 5′ UTRs relative to coding sequences upon DDX3 depletion (Figure 2C), especially in mRNAs that show translational downregulation. As an example, *HMBS* ribosome occupancy is shown in Figure 2D, which showed changes in ribosome density in its 5′ UTR and therefore may be regulated by upstream ORF (uORF) translation.

### DDX3 crosslinks to ribosomal RNA and 5′ UTRs

**Figure 3.**
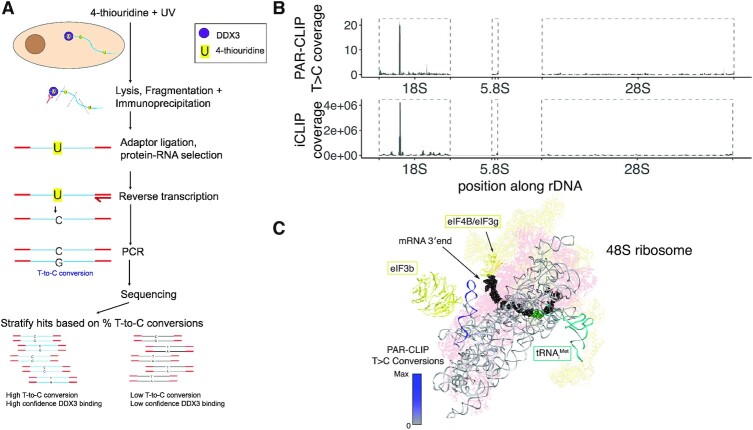
DDX3 binding sites on rRNA identified by PAR-CLIP. (**A**) A workflow of the PAR-CLIP experiment. (**B**) PAR-CLIP T>C conversion locations on human rDNA (top) compared to iCLIP coverage from Oh *et al.* 2016 (bottom). Boxed regions refer to processed rRNA transcripts. (**C**) PAR-CLIP T>C conversion density on the 18S rRNA is visualized from gray to blue on the structure of the 48S ribosome (PDB 6FEC). The peak in (B) is contained in the helix in the upper left in (C), which is h16 of rRNA. Yellow: translation factors; blue–gray: rRNA; pink: ribosomal proteins.

The above ribosome profiling and RNA-seq experiments identified the set of transcripts that are affected by DDX3 depletion, but these transcripts could be affected by direct or indirect mechanisms. Therefore, to better define the set of transcripts that are direct targets of DDX3, we measured DDX3 binding sites with high specificity using PAR-CLIP (Figure 3A, Supplementary Figure S1A). Previous work using a complementary method (iCLIP) to measure DDX3 binding sites identified 5′ UTR and ribosomal RNA binding. Curiously, even though DDX3 is thought to regulate translation initiation, binding was also identified in coding sequences and 3′ UTRs (3,12). Here, we used the additional specificity afforded by T>C transitions induced by protein adducts on crosslinked uridine residues in PAR-CLIP to refine DDX3 binding sites across the transcriptome (46).

High-throughput sequencing of RNA fragments crosslinked to DDX3 identified a binding site for DDX3 on the 18S ribosomal RNA (Figure 3B; visualized on the structure (56). It is possible that these rRNA reads could arise from nonspecific interactions between RNA binding proteins and the highly abundant rRNA. However, while there were many rRNA fragments sequenced following PAR-CLIP, there was only one site with high-confidence T>C transitions, spanning nucleotides 527–553 in the 18S rRNA (Figure 3B, Supplementary Figure S4A). This site maps to helix 16 of the 18S rRNA, similar to where Ded1 crosslinks to 18S rRNA in yeast, and does not contain post-transcriptionally modified rRNA nucleotides (1,57). Helix 16 (h16) is on the small ribosomal subunit facing incoming mRNA, which might provide DDX3 access to resolve mRNA secondary structures to facilitate inspection by the scanning 43S complex (Figure 3C). The crosslink site on h16 is just opposite an RRM domain that has been assigned as eIF4B, another factor crucial in ribosome recruitment and scanning (56,58,59). This is consistent with observations that eIF4B and Ded1 cooperate in translation initiation on mRNAs (58). Recently, it has been proposed that this RRM domain may belong to eIF3g, another translation initiation factor (60), which is consistent with a reported interaction between eIF3 and DDX3 (26).

**Figure 4.**
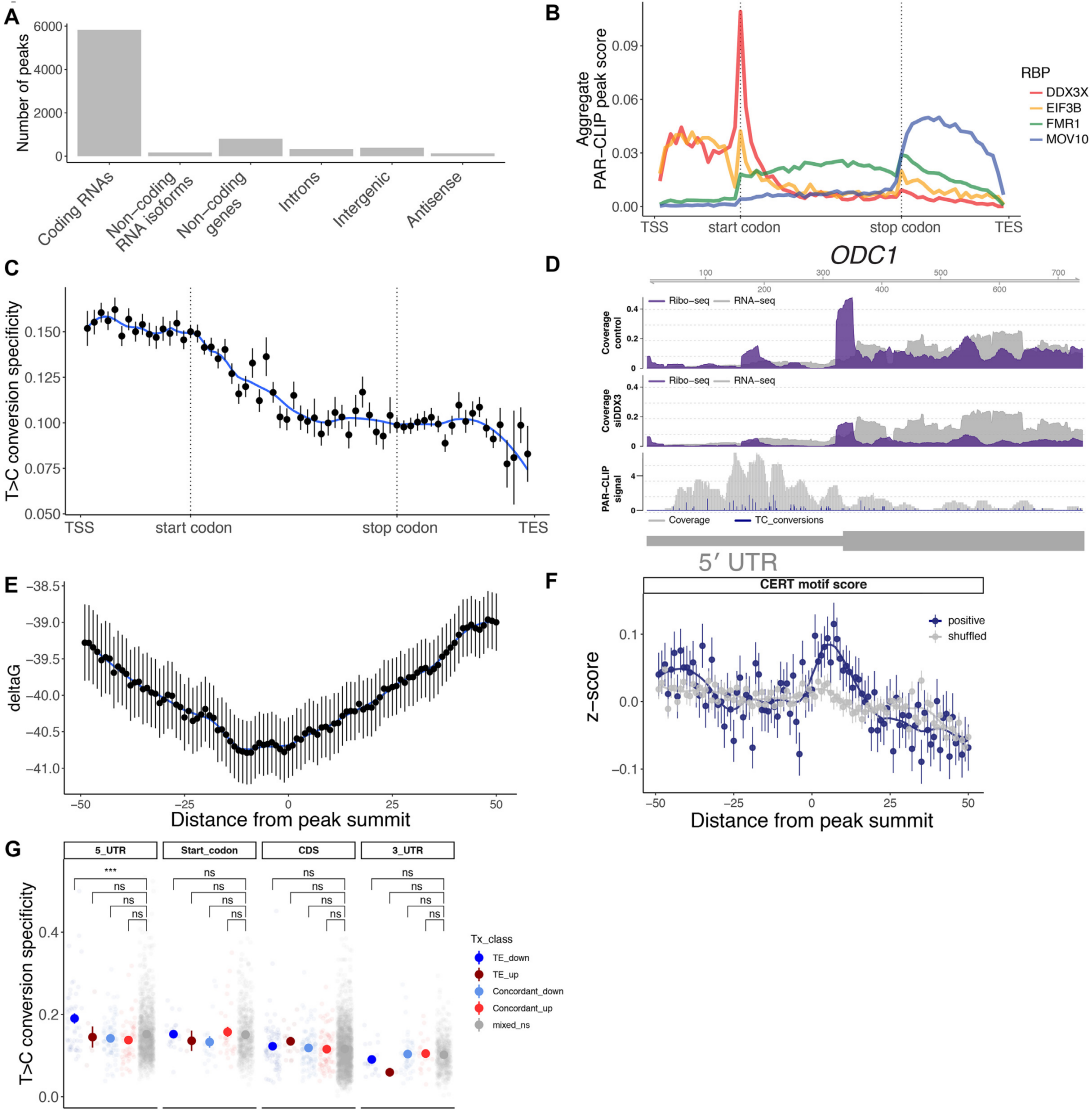
DDX3 binds to structured 5′ leaders of mRNA. (**A**) Sum of the DDX3 PAR-CLIP peaks mapping on different gene types and regions. (**B**) A metagene plot of DDX3 PAR-CLIP across all genes and PAR-CLIP data from other RNA binding proteins. eIF3b is a canonical initiation factor, FMR1 binds elongating ribosomes, and MOV10 is a 3′ UTR binding factor. TSS: transcription start site, TES: transcription end site. (**C**) T>C conversion specificity averaged across all PAR-CLIP peaks across indicated mRNA regions as in (B). (**D**) PAR-CLIP, ribosome profiling, and RNA-seq across part of the *ODC1* gene. Blue peaks in the PAR-CLIP track indicate T>C conversion events. (**E**) RNA structure in a window of 100 nucleotides around PAR-CLIP peak summits in 5′ UTRs. (**F**) CERT motif scores averaged across PAR-CLIP peak summits in 5′ UTRs or for shuffled positions. (**G**) PAR-CLIP T>C conversion specificity in transcript regions in indicated gene sets from Figure 1.

In addition to ribosomal RNA binding, we found that DDX3 interacts primarily with coding transcripts (Figure 4A, Supplementary Table S2). To identify where DDX3 binds mRNAs, we aggregated peaks across all expressed transcripts in a metagene analysis. We found that DDX3 primarily contacts transcript 5′ UTRs, with a small number of reads mapping in the coding sequence and 3′ UTR (Figure 4B). A large peak was also observed at the start codon, which could reflect kinetic pausing during subunit joining while DDX3 is still bound to the initiating ribosome (61). We used available CLIP data to compare the binding pattern of DDX3 to other known mRNA-binding proteins (39). We selected three RNA-binding proteins to compare to: eIF3b is a member of the multi-subunit initiation factor eIF3 (62), FMR1 interacts with elongating ribosomes (63), and MOV10 is involved in 3′ UTR-mediated mRNA decay (64,65). The binding pattern of DDX3 most closely resembles the initiation factor eIF3b (Figure 4B). We detected some DDX3 binding within coding sequences and even 3′ UTRs, which could arise from background signal, or alternative binding modes. PAR-CLIP indicated that DDX3 binds abundant mRNAs (Supplementary Figure S4B), including ribosomal protein genes. While this observation can provide insights into the molecular functions of DDX3, it might reflect a limit in the sensitivity of our PAR-CLIP data. Therefore, we decided to investigate PAR-CLIP binding patterns which are not strongly confounded by expression levels. We used the frequency of T>C transitions at each site as a measure of the specificity of protein-RNA interactions (66). High specificity crosslinks with frequent T>C transitions resided most often in 5′ UTRs (Figure 4C), as also shown in a translationally-regulated transcript such as *ODC1* (Figure 4D). Confirmation of this binding pattern comes from an independent assay of protein–RNA interaction, as measured by enhanced CLIP (eCLIP) (Supplementary Figure S4C; 67).

Next, we sought to describe the mRNA regions with enriched DDX3 binding. DEAD-box RNA helicases engage RNA by recognizing structural elements with poorly defined sequence context (68), which hinders the ability to extract meaningful sequence motifs from CLIP data. To investigate this phenomenon, we performed *de novo* motif finding on PAR-CLIP peaks in HEK 293 cells from the POSTAR2 repository (39; Materials and Methods). As expected, motifs extracted from different RBPs showed different degrees of specificity (Supplementary Figure S4D). DDX3 motifs, together with motifs from translation initiation factors and other ribosome interactors, showed poor significance when compared to other RBPs with more defined sequence specificity (such as members of cleavage and polyadenylation machinery, or known 3′UTR binders). This result called for a more targeted analysis of DDX3 binding sites. By investigating the sequence-structure context around mRNA peaks in 5′ UTRs, we observed that DDX3 binding sites by PAR-CLIP reside in highly structured regions (Figure 4E). We observed a higher guanine content (accompanied by predicted G-quadruplexes; Supplementary Figures S4E, F) upstream of the binding site; downstream of the peak summit, we detected high GC content resembling the CERT motif (Figure 4F), a regulatory motif highly predictive of DDX3-mediated translation regulation (Figure 2A). Moreover, we observed increased T>C conversion specificity in the 5′ UTR of transcripts whose translation decreases upon DDX3 depletion (Figure 4G), indicative of a possibly stronger protein-RNA association at those regions. This binding pattern, combined with the observation that DDX3 co-fractionates with both polysomes and initiation complexes, suggests that DDX3 acts with the 40S during the process of translation initiation (2,69–71). Taken together, we conclude that the DDX3 binds and regulates the translation of poorly translated mRNAs exhibiting complex sequence-structure features in their 5′UTRs.

### Identifying translation changes caused by DDX3 mutations

*De novo* genetic variants in *DDX3X* cause developmental delay and intellectual disability in DDX3X-syndrome (13–16). Interestingly, patients carrying inactivating point mutations in DDX3 display more severe clinical symptoms than patients with truncating mutations (16). Point mutations in DDX3 associated with medulloblastoma are dominant negative and act by preventing enzyme closure of DDX3 towards the high-RNA-affinity ATP-bound state (6–11). This suggests there may be different effects on translation between depletion of DDX3 and inhibition or expression of an inactive mutant.

**Figure 5.**
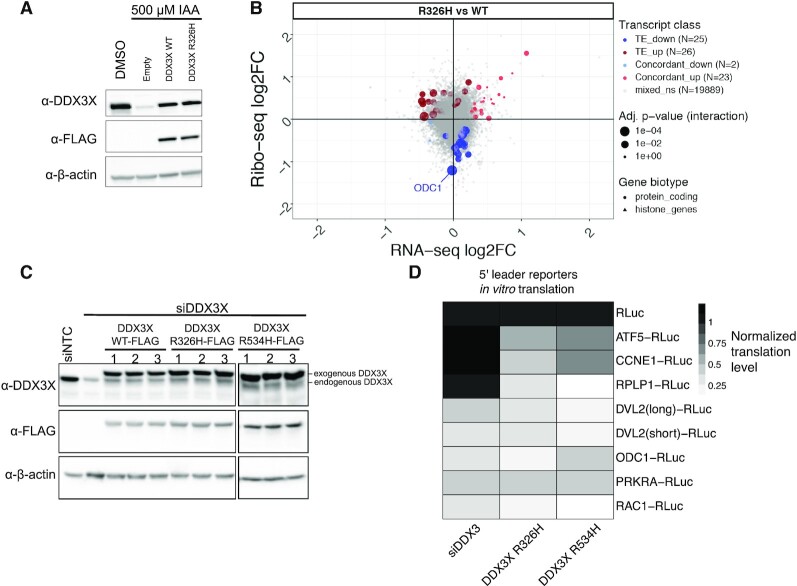
Translation changes caused by R326H mutant DDX3. (**A**) Western blots of degron DDX3 cells treated with IAA and transfected with empty vector or the indicated constructs. (**B**) Ribosome profiling and RNA-seq of DDX3 degron cells treated with IAA and transfected with either DDX3 wild-type or R326H mutant. (**C**) Western blots of cells treated with siDDX3 and transfected with the indicated constructs. (**D**) *In vitro* translation performed with indicated reporter RNAs as in Figure 1 in the lysates from panel (C). Reporter sequences are in Supplementary Table S4.

To test the effect of mutants in DDX3 on translation, we transfected cells with plasmids containing wild-type or mutant DDX3 proteins after auxin-induced degradation of endogenous DDX3, switching expression from the wild-type sequence to an allele of interest (Figure 5A). We used this system to define acute changes to translation caused by DDX3 mutations without allowing the cells to adapt to the presence of inactive DDX3 alleles, which may also be lethal. We measured genome-wide translation changes caused by DDX3 mutants using ribosome profiling (Supplementary Table S1). A collection of genes related to the double-stranded DNA response were upregulated at the level of RNA abundance, likely due to differences in transfected DNA amounts (Figure 5B, upper right). Broadly, we noticed higher variability in in ribosome occupancy changes (Figure 5B, y-axis) than RNA level changes (Figure 5B, x-axis) when compared to the siDDX3 experiments (Figure 1C), suggesting that one functional difference between mutant and knockdown might involve regulation of RNA steady-state levels. Among the few regulated mRNAs, we observed a robust downregulation of *ODC1* translation, which was directly bound by DDX3 (Figure 4D) and strongly downregulated in the DDX3 depletion experiment (Figure 1C).

To further test the difference between knockdown and mutant DDX3 at the level of individual 5′ UTRs, we made *in vitro* translation extracts with wild-type DDX3, R326H DDX3, or R534H DDX3 and measured translation of a panel of reporters (Figure 5C; (3)). Broadly, we found two classes of reporter RNAs (Figure 5D; Supplementary Figure S5A, B). One class, including *ODC1, PRKRA, RAC1* and *DVL2* isoforms decreased in translation in all tested perturbations to DDX3. Another class, including *ATF5, CCNE1* and *RPLP1* selectively decreased in translation in mutant DDX3 extracts but not in knockdown extracts. We sought to test chemical inhibition of DDX3, as it functionally mimics a dominant negative mutation by blocking ATP binding. We were unable to inhibit translation in a DDX3-dependent manner using RK-33 (72), and instead found that it acted as a general translation inhibitor (Supplementary Figure S5C). Taken together, we found that DDX3 sensitivity for translation is preserved in translation extracts and that depletion of DDX3 appears to have different outcomes on translation than inhibition or dominant negative variants.

## DISCUSSION

**Figure 6.**
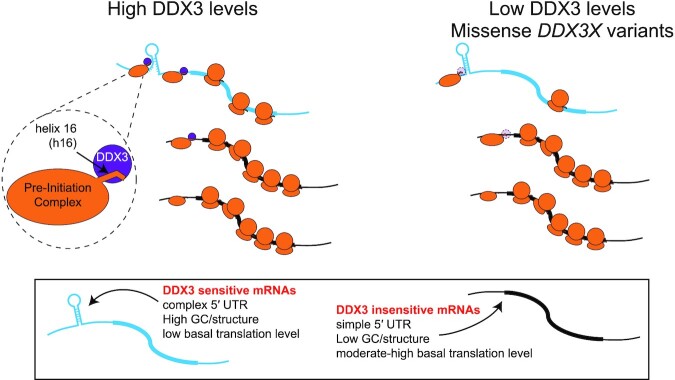
A model of DDX3 in translational control. DDX3 binds to the small subunit via helix 16 (h16). Transcripts that are poorly translated in normal cells and that harbor increased intramolecular RNA structure (blue transcript; left) are sensitive to DDX3 depletion (right). Other mRNAs that are initially highly translated (black transcript) are unaffected.

*DDX3X* is an essential human gene that is altered in diverse diseases. Here, we use a set of transcriptomics approaches, machine learning and biochemistry to show that DDX3 regulates a subset of the human transcriptome, likely through resolving RNA structures in 5′UTRs (Figure 6). Reporter experiments show that 5′ UTRs are sufficient to confer DDX3 sensitivity onto unrelated coding sequences. We conclude that DDX3 affects translation initiation through transcript 5′ UTRs. Our data suggest that the major role of DDX3 is in translation initiation and reveal translation differences between mutated and haploinsufficient DDX3 expression.

We identified binding between DDX3 and helix 16 (h16) on the human 40S ribosome. This is similar to binding sites identified previously using other CLIP approaches (1,3,12), confirmed here using T>C transitions defined by PAR-CLIP. Interestingly, histone mRNAs showed sustained translation levels upon DDX3 knockdown (Figure 1C). However, histone mRNAs represent a peculiar category of transcripts, often containing short UTRs, few introns, highly repetitive sequences and the lack a poly-A tail. The quantitative contribution of some of these features have been resolved by our regression approach, with good agreement between the lasso and the random forest models. However, we believe that more tailored approaches are needed to precisely investigate regulation of specific classes of mRNAs. For instance, histone mRNA translation has been recently shown to be dependent on mRNA binding to the rRNA h16 helix (73). Our data suggests that there might be competition for h16 between DDX3 and histone mRNAs, and their translation increases upon DDX3 knockdown due to increased accessibility to h16. The set of mRNAs that require h16 for their translation will be an interesting direction to pursue in the future.

Despite primarily affecting translation, some genes exhibited changes in steady-state RNA abundance upon DDX3 depletion. RNA-level changes could be mediated by indirect effects of a DDX3-dependent translation target, or reflect additional mechanisms of post-transcriptional gene regulation, possibly mediated by RNA structural features, codon composition, or other RNA decay pathways (74). Moreover, DDX3 is an important factor in stress-granule complexes (75), and possibly involved in granule-specific regulation of mRNA metabolism. Interestingly, we observed more RNA-level changes in DDX3-depletion experiments (Figure 1C and Supplementary Figure S2B) than in experiments using a mutation in DDX3 (Figure 5B). Potential interactions between DDX3 regulation of both ribosome occupancy and RNA levels will be further explored in future studies.

DDX3 is an abundant protein, with approximately 1.4 million copies per HeLa cell (76), or about half the abundance of ribosomes (77). We have interpreted data in this work by hypothesizing that DDX3 is functioning *in cis* by binding to the 40S ribosome and facilitating translation initiation on the associated mRNA (Figure 6). It is also possible that DDX3, alone or in combination with other DEAD-box proteins like eIF4A, functions *in trans* by activating an mRNA prior to 43S complex loading. Future work defining the binding site of DDX3 on the ribosome could enable separation of *cis* and *trans* functions to test these two models, although we note that the functional consequences of DDX3 depletion we have observed here are independent of its functioning in *cis* or *trans*.

DDX3 is altered in numerous human diseases, including cancers and developmental disorders (5). Some diseases are characterized by missense variants (8–11), while others involve predominantly nonsense or frameshift variants (17,18), and still others present with a mixture of variant types (13–16). Our work suggests that variants in DDX3 that deplete protein levels may result in different translation changes than inactivating missense variants. We attempted to directly compare translational changes upon DDX3 depletion identified in this work with previous expression of mutant DDX3 but stopped due to confounding variability in biological sample and library preparation and sequencing protocols. Defining how different mutation types in DDX3 affect gene expression, the underlying molecular mechanisms, and potential therapeutic interventions is an intriguing direction for the future.

## DATA AVAILABILITY

Processed datasets and code to reproduce the main figures can be found here: https://github.com/lcalviell/DDX3X_RPCLIP.

Sequencing data can be retrieved using GEO accession numbers GSE125114 and GSE157063.

## Supplementary Material

gkab287_Supplemental_Files
