# Supplementary material for: DDX3 depletion represses translation of mRNAs with complex 5′ UTRs
Source: Nucleic Acids Res. 2021 Apr 27;49(9):5336–50. doi: 10.1093/nar/gkab287 (PMC8136831; doi:10.1093/nar/gkab287)
Supplement: gkab287_Supplemental_Files [file gkab287_supplemental_files.zip › supplemental information.pdf]

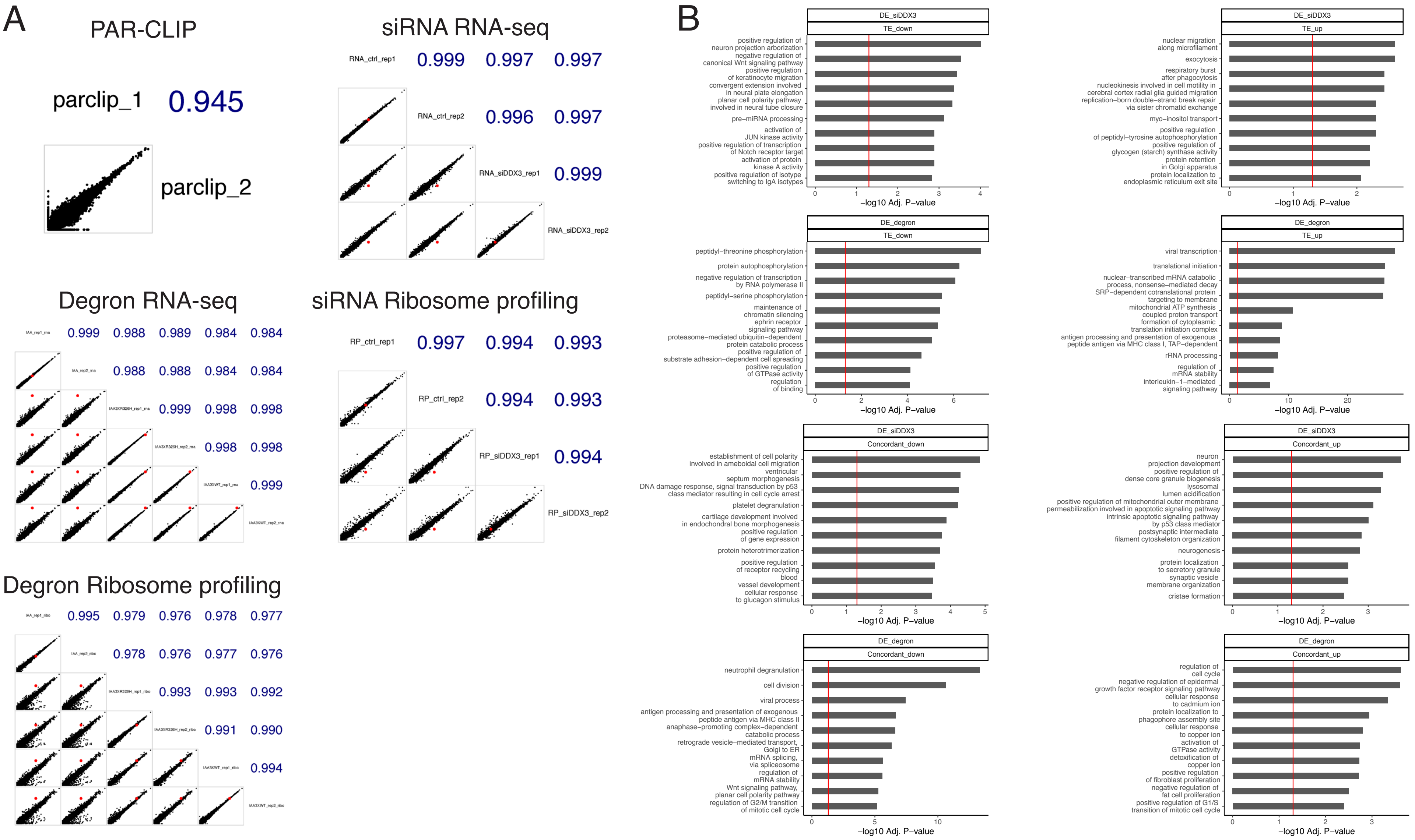

Figure S1: Related to Figure 1 high-throughput sequencing analyses.
(A) Replicate correlations for indicated high-throughput sequencing experiments. X and Y axes are TPM values with DDX3X shown as a red dot. (B) Gene ontology analyses for the siDDX3 and degron ribosome profiling experiments. Note that the siDDX3 experiments were in HEK 293T cells while the degron experiments were in HCT 116. Red line at P = 0.05.

Calviello, Venkataramanan *et al*. Figure S1

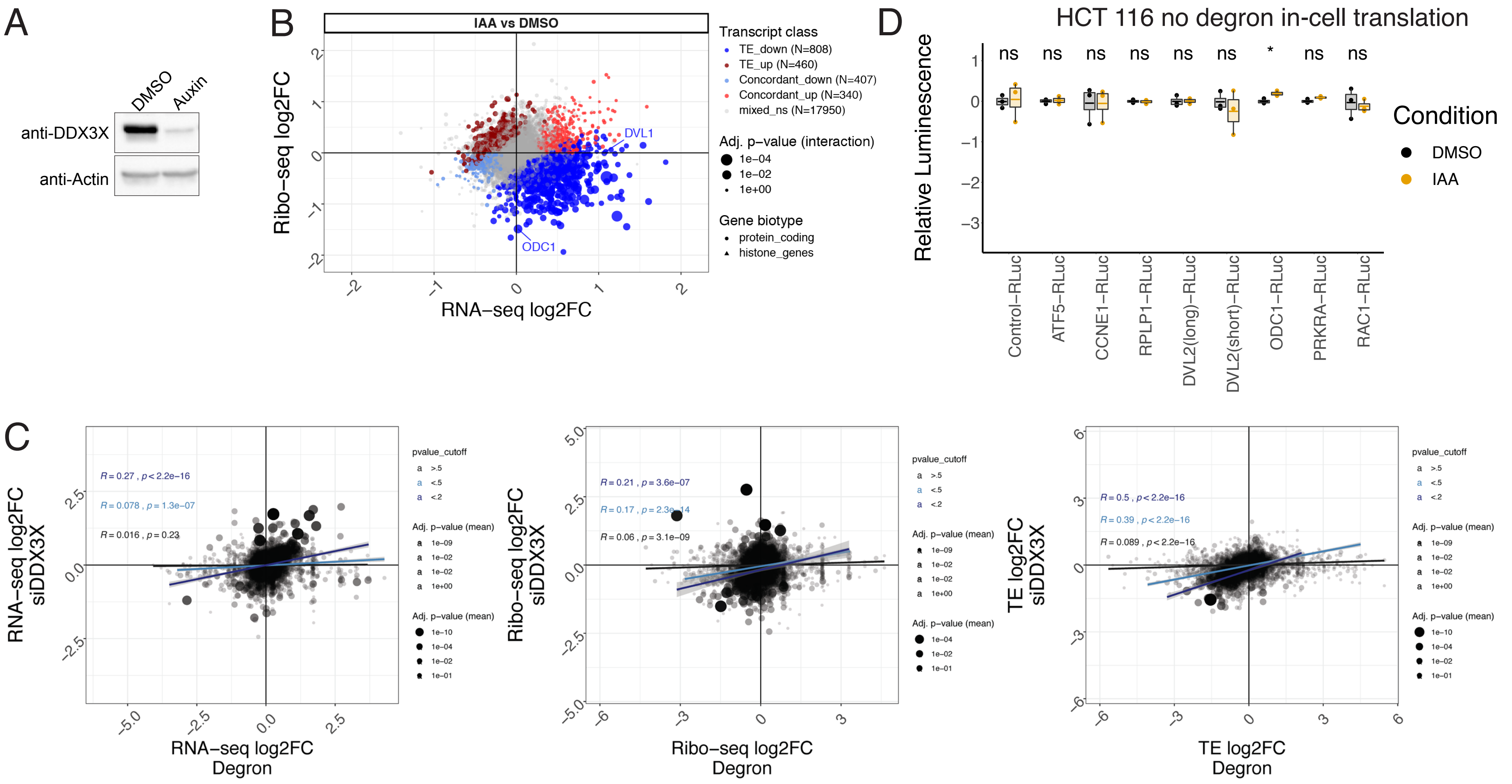

Figure S2: Related to Figure 1 ribosome profiling.
(A) Western blot of the DDX3 degron cells treated with IAA (Auxin). (B) Ribosome profiling and RNA-seq fold changes in DDX3 degron (IAA) versus control (DMSO) cells. (C) RNA levels from RNA-seq (left), ribosome protected footprint levels from ribosome profiling (center), and translation efficiency (TE; right) were calculated for all genes in the siDDX3 or DDX3-degron experiments and are plotted. All values plotted are log2 fold change. P values for each panel are calculated using DESeq2 as described in Methods. Correlations are shown at three separate p value cutoffs; note genes with more significant TE changes are more highly correlated between siDDX3 and the degron. (D) In-cell translation with indicated reporter RNAs that were in vitro transcribed and transfected into HCT 116 cells not containing a DDX3X degron tag. Relative luminscence between Renilla reporters and Firefly controls indicated.

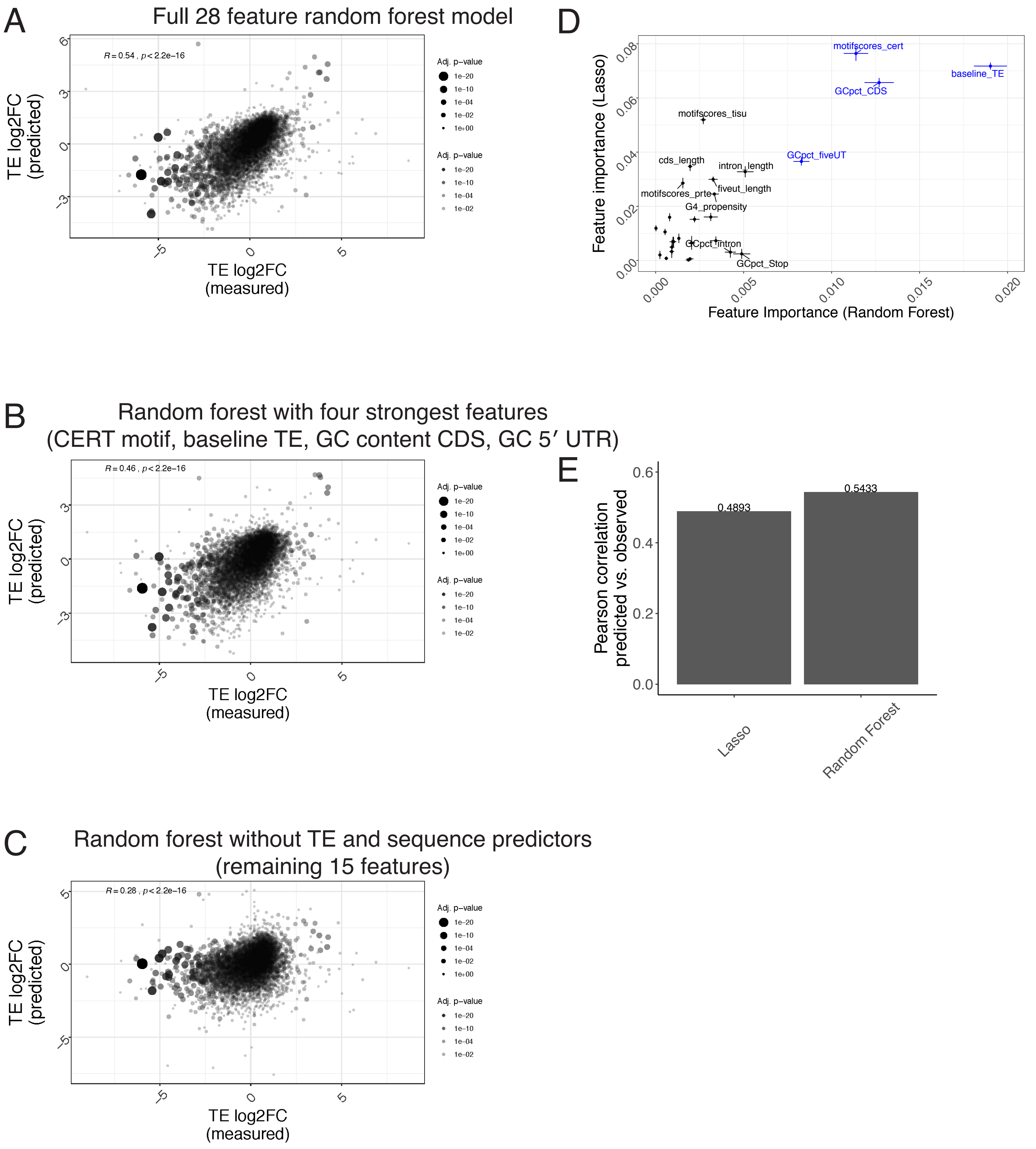

Figure S3: Related to Figure 2 ribosome profiling feature analysis
(A) Correlation between the predicted and observed TE changes, using A) a full Random Forest model including all features shown in Figure 2, a B) reduced model with relevant features, or C) less informative features (no baseline TE or sequence features). (D) Comparison of feature importance derived from Lasso regression versus a Random Forest model. (E) Pearson correlations between predicted and observed TE changes using lasso or random forest.

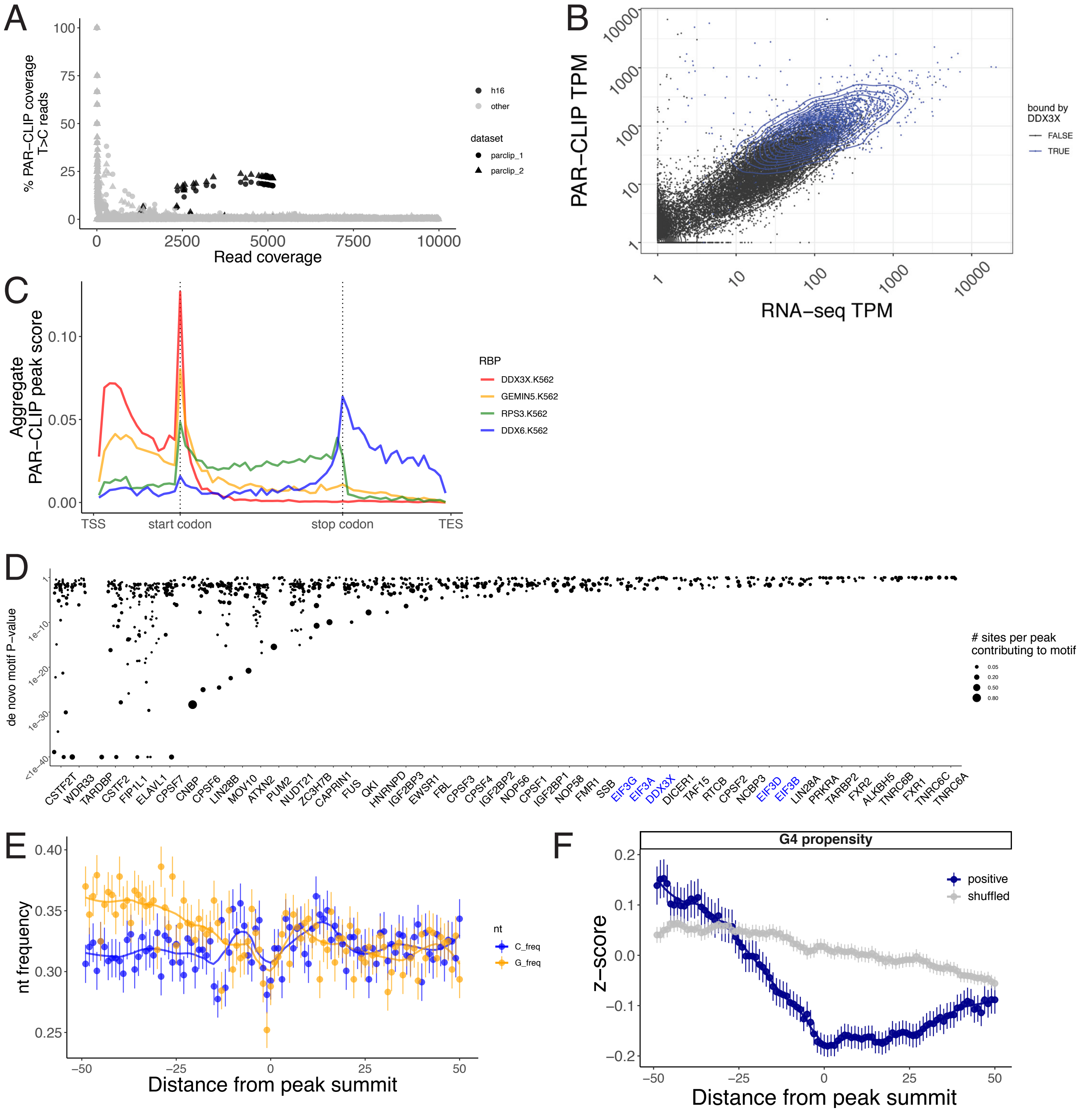

Figure S4: Related to Figure 4 - PAR-CLIP.

(A) PAR-CLIP T>C conversion coverage at each rRNA position versus its read coverage for both replicates. Helix 16 (h16) reads have both high T>C coverage and overall read coverage. (B) PAR-CLIP TPM versus RNA-seq TPM with called DDX3X targets in blue. (C) Metagene analyses for eCLIP datasets from indicated proteins across indicated transcript regions. eCLIP experiments were performed in K562 cells. (D) Motif p-values from STREME for the 50 indicated RNA-binding proteins with translation factors in blue. Each point represents a detected motif. (E) Guanosine and cytosine frequency averaged across all PAR-CLIP peak summit locations in 5′UTRs. (F) Predicted RNA G-quadruplex propensity of PAR-CLIP sites in 5′UTRs versus shuffled.

Calviello, Venkataramanan *et al*. Figure S4

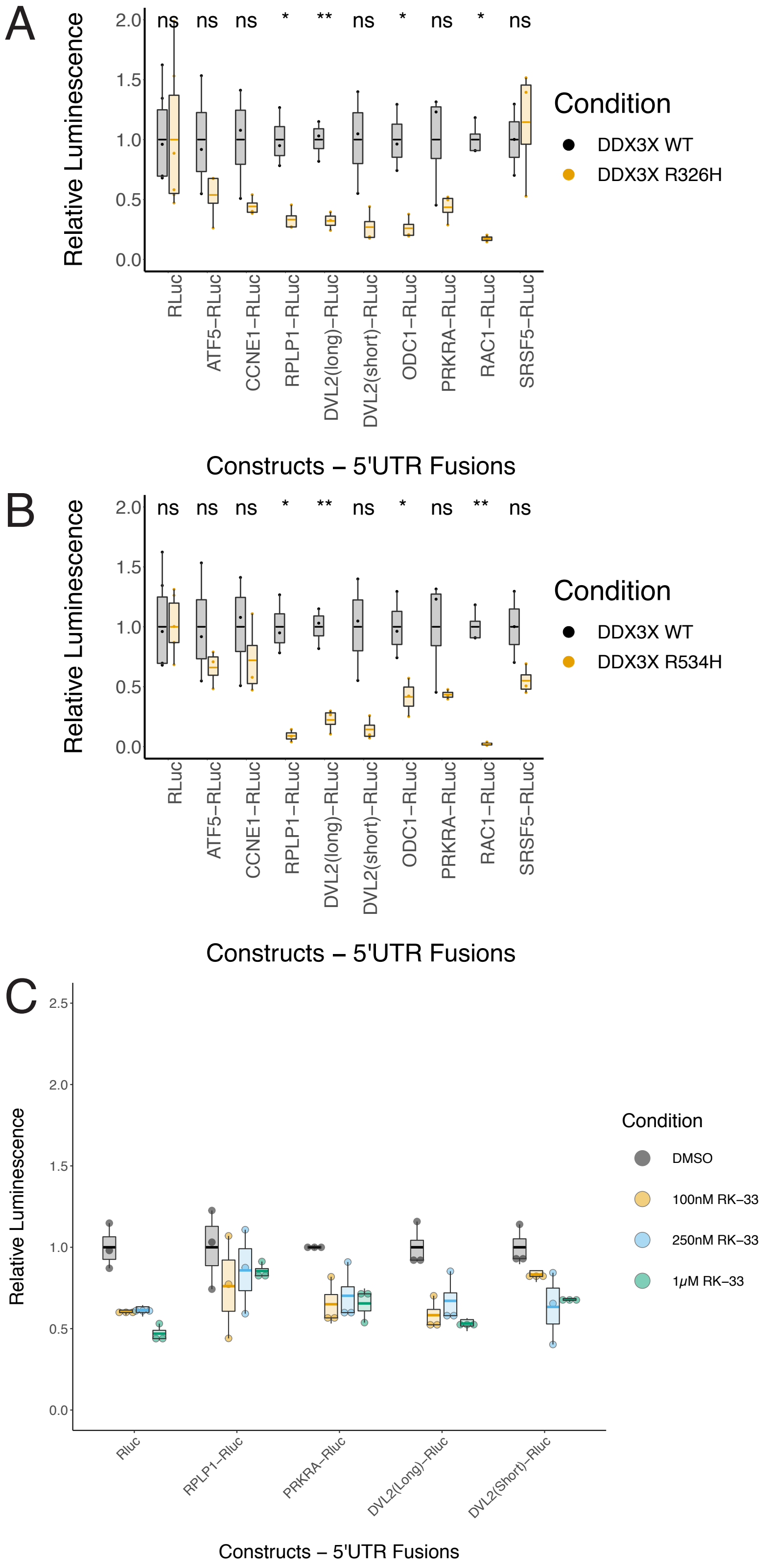

Figure S5: Related to Figure 5 in vitro translation.
(A, B) In vitro translation performed with Renilla luciferase fused to the various 5′ leaders indicated. siRNA knockdown of DDX3 complemented with R326H (A) or R534H (B) mutant DDX3X, respectively, compared against siRNA knockdown of DDX3 complemented with wild-type DDX3. (C) In vitro translation with the small molecule RK-33 reveals DDX3-independent inhibition of mRNA translation (compare Rluc control to DDX3-specific 5′ UTR reporters).
