# Supplementary material for: DDX3 depletion represses translation of mRNAs with complex 5′ UTRs
Source: Nucleic Acids Res. 2021 Apr 27;49(9):5336–50. doi: 10.1093/nar/gkab287 (PMC8136831; doi:10.1093/nar/gkab287)
Supplement: gkab287_Supplemental_Files [file gkab287_supplemental_files.zip › Table S4-sequences of 5utr reporters-v2.docx]

Statistics of reporter 5′ UTRs:

Sequences of reporter 5′ UTRs:

>control

CTAGCCACC

>ATF5_ENST00000595125

ATTTGGGAGAGTGCTGTGACTCATGCTGGACTCTAACCCACGAGGGTTTCTCAGAGTCAGCAGCTGGGGGATGAAGAAGTGAAAAGTGACTGGCAGGAAATTCTGCAAGCAAGGAAAGGGAAAGAGAAATGAACTGGTGCAGGTCTGCGGGAAGAGAATGAGGCTGGATCCTCAAAATCACAGGAGGAAGCAGGCCCAGACCTCAGAGGCAGAAAGAGAAAGAAACCAGAGCTTAGAGTCAGGAGGAGGAAACCAGACCCCGGAGCCACAAGGAGAGGGCTGGATCCCCGGCTCAGAGGGAAGAGTGTCGCCGCCTCTGCCTGCGTAGCCCCGGCCATGGCTCTGTAGCCTCGACCCCTTTGTGCCCCCGGCCCGTCTCCGCGCTCACCACGCCTGCGCTCTCCGCTCCCACCTTCTTTCTTCAGCCGAGGCCGCCGCCGCCTCTCCTTGCTGCAGCCATGGAGTCTTCCACTTTCGCCTTGGTGCCTGTCTTCGCCCACCTGAGCATCCTCCAGAGCCTCGTGCCAGCTGCTGGTGCAGCCTCTCCTGTTGCCATCAGTGCCCAGCACCTGTGCTACAGCC

>RPLP1_ENST00000260379

TGGACACATAAGAGGCTGCGTATAGGCGCGAGAGCCCCTTTCCTCAGCTGCCGCCAAGGTGCTCGGTCCTTCCGAGGAAGCTAAGGCTGCGTTGGGGTGAGGCCCTCACTTCATCCGGCGACTAGCACCGCGTCCGGCAGCGCCAGCCCTACACTCGCCCGCGCC

>DVL2_short_no_accession

TCGCACCCCGCGGCCCGCCCCCCGCCGCCACCCTCGCAGATCCGTGCTTTTTCCCCTTTGCTTCTCTCCCGTACTGGGTCAGTCCTGTCCGCGCTCGCGCGTCGGTTTGCGGGTGTGCGCAGGCGCGGCAGGGGCCATTAGCCCTTTGGGTGGGCGGTGGAGCCCGGGAGCGCGCGGGCGAGACC

>DVL2_long_ENST00000005340

TTAAGTCACGTGACATGAGGAGAGGTGGGCGGGTACCTGGAGGAAGCTCGCGGCGTCGGTGGCGGTGGCGCGCGGCGGCCGCTGAGACCGGGGCTTTGAGTCGCACCCCGCGGCCCGCCCCCCGCCGCCACCCTCGCAGATCCGTGCTTTTTCCCCTTTGCTTCTCTCCCGTACTGGGTCAGTCCTGTCCGCGCTCGCGCGTCGGTTTGCGGGTGTGCGCAGGCGCGGCAGGGGCCATTAGCCCTTTGGGTGGGCGGTGGAGCCCGGGAGCGCGCGGGCGAGACC

>PRKRA_ENST00000325748

GCGAGGGGGCGTAGCCGGAGCTACGGCACCAAGGCTCCGCCCCCACCCTGCCTGCCCCCTCGCTGGAGCAACGCAAGCAGGAGGCGGGGGAGTCGGAGGAGGTGGCGGCGCTGGAGCTCCTCCCGGGGACCAGCGACCCGGGGAGCGAGCACGTCGCTCCGCACCGCTCTTCCTCCAGCCGCTGAGCCGTCCCTTCTCGCC

>RAC1_ENST00000356142

AGTTTTCCTCAGCTTTGGGTGGTGGCCGCTGCCGGGCATCGGCTTCCAGTCCGCGGAGGGCGAGGCGGCGTGGACAGCGGCCCCGGCACCCAGCGCCCCGCCGCCCGCAAGCCGCGCGCCCGTCCGCCGCGCCCCGAGCCCGCCGCTTCCTATCTCAGCGCCCTGCCGCCGCCGCCGCGGCCCAGCGAGCGGCCCTG

>ODC1_ENST00000234111

GACGTCGGCCCGCCGGCGCCCCACCAGCTCCGCGCGGGCCCGGGTTGGCCACCGCCGGGCCCCCGCCCCTCCCCCGGCGGTGTCCCGGCCGGAACCGATCGTGGCTGGTTTGAGCTGGTGCGTCTCCATGGCGACCCGCCGGTGCTATAAGTAGGGAGCGGCGTGCCGTGGGGCTTTGTCAGTCCCTCCTGTAGCCGCCGCCGCCGCCGCCCGCCGCCCCTCTGCCAGCAGCTCCGGCGCCACCTCGGGCCGGCGTCTCCGGCGGGCGGGAGCCAGGCGCTGACGGGCGCGGCGGGGGCGGCCGAGCGCTCCTGCGGCTGCGACTCAGGCTCCGGCGTCTGCGCTTCCCCATGGGGCTGGCCTGCGGCGCCTGGGCGCTCTGAGATTGTCACTGCTGTTCCAAGGGCACACGCAGAGGGATTTGGAATTCCTGGAGAGTTGCCTTTGTGAGAAGCTGGAAATATTTCTTTCAATTCCATCTCTTAGTTTTCCATAGGAACATCAAGAAATC

>CCNE1_ENST00000262643

GAGGGGCTGGGAGCCGCGGCGGGGCGGTGCGAGGGCGGGCCGGGGCCGGTTCCGCGCGCAGGGATTTTAAATGTCCCGCTCTGAGCCGGGCGCAGGAGCAGCCGGCGCGGCCGCCAGCGCGGTGTAGGGGGCAGGCGCGGATCCCGCCACCGCCGCGCGCTCGGCCCGCCGACTCCCGGCGCCGCCGCCGCCACTGCCGTCGCCGCCGCCGCCTGCCGGGACTGGAGCGCGCCGTCCGCCGCGGACAAGACCCTGGCCTCAGGCCGGAGCAGCCCCATC
